# Supplementary material for: Kombucha-Derived Cellulose Non-wovens: Growth Optimization, Mechanics, and Recycling
Source: bioRxiv. 2026 Jun 12:2026.06.09.730694. Preprint. [Version 1] doi: 10.64898/2026.06.09.730694 (PMC13277940; doi:10.64898/2026.06.09.730694)
Supplement: Supplementary file 1 [file media-1.pdf]

Supporting Information

Kombucha-Derived Cellulose Non-wovens: Growth Optimization, Mechanics, and Recycling

*Luying He[1], Julia E. Lopez[2] and Jessica D. Schiffman[1,2]**

[1]Materials Science and Engineering Graduate Program, University of Massachusetts Amherst, Amherst, Massachusetts 01003, United States

[2]Department of Chemical and Biomolecular Engineering, University of Massachusetts Amherst, Amherst, Massachusetts 01003

* Corresponding author: Jessica D. Schiffman, Email: schiffman@umass.edu

Supplementary Methods: Mechanical Testing of Kombucha-Derived Non-Wovens.

Oscillatory Rheological Method and Data Analysis. The amplitude (strain) sweep identified the linear viscoelastic region (LVR). The reported LVR limit corresponds to the strain at which G′ deviated by ≤5% from its plateau. Frequency sweeps were then run within the LVR (0.1 to 100 rad s^{-1} at a fixed strain ≤ the LVR limit for each state). G′ at 1 rad s^{-1} is reported from the frequency sweep. Values summarize independent specimens per state.

Uniaxial Tensile Method and Data Analysis. Young's modulus, E, was determined by linear regression of the initial linear regime of the engineering stress-strain response. Ultimate tensile strength (UTS), σ_{max}, was defined as the maximum engineering stress reached prior to failure, and elongation at break, ε_b, was taken as the engineering strain at failure. The strain energy density to fracture, W, was defined as the area under the engineering stress-strain curve up to the point of failure as **Equation S1**:

$$W = \int_0^{\varepsilon_b} \sigma(\varepsilon)\, d\varepsilon \quad \textbf{(S1)}$$

where W is the strain energy density to fracture, $\sigma(\varepsilon)$ is the engineering stress at strain ε, ε is the engineering strain, and ε_b is the strain at break. Numerical integration was performed using the trapezoidal rule. The fracture surface energy, G, defined as the energy dissipated per unit newly created fracture surface area, was estimated using **Equation S2**:

$$G = tW \quad \textbf{(S2)}$$

where G is the fracture surface energy, t is the sample thickness, and W is the strain energy density to fracture. This approach assumes uniform energy dissipation across the fracture plane and follows common practice for estimating fracture energies of soft and fibrous materials from tensile measurements.

Table S1. Influence of Culture Variables on the Thickness of Kombucha-Derived Non-Wovens.

**Culture variables**		**Thickness** (mm)	**Normalized Ratio†**
**Inoculation** ($L \cdot L^{-1}$)	0.01	0.096 ±0.1	0.00
	0.02	0.064 ±0.04	-0.33
	0.05	0.36 ±0.06	2.75
	0.1	1.769 ±0.1	17.43
**Sucrose** ($g \cdot L^{-1}$)	0	0.003 ±0.001	0.00
	50	0.76 ±0.2	252.33
	100	2.08 ±0.2	692.33
	150	2.34 ±0.3	779.00
**Tea leaves** ($g \cdot L^{-1}$)	5	0.36 ±0.1	0.00
	10	0.69 ±0.04	0.92
	15	1.11 ±0.5	2.08
	20	0.95 ±0.1	1.64
**Temperature** (°C)	20	1.25 ±0.1	0.00
	25	1.07 ±0.05	-0.14
	30	1.68 ±0.2	0.34
	35	0.13 ±0.06	-0.90
**pH Value**	3	0.20 ±0.07	0.00
	4	1.35 ± 0.1	5.75
	5	0.08 ±0.02	-0.60
	6	0.01 ±0.0	-0.95

†Calculated using **Equation 2**

Table S2. Thickness of Non-Wovens Under Different Fermentation, Drying, and Rehydration Conditions.

**Sample Name**	**Fermentation Time** (days)	**Thickness** (mm)	**Normalized Ratio***
Wet	14	1.24 ±0.1	0.00
Thin	7	0.39 ±0.1	-68.55
Thick	21	2.52 ±0.4	103.23
Oven-dried	14	0.26 ±0.1	-79.03
Lyophilized	14	0.27 ±0.04	-78.23
Rehydrated Oven-dried	14	1.04 ±0.1	-16.13
Rehydrated Lyophilized	14	1.05 ±0.08	-15.32

*As calculated using **Equation 2**

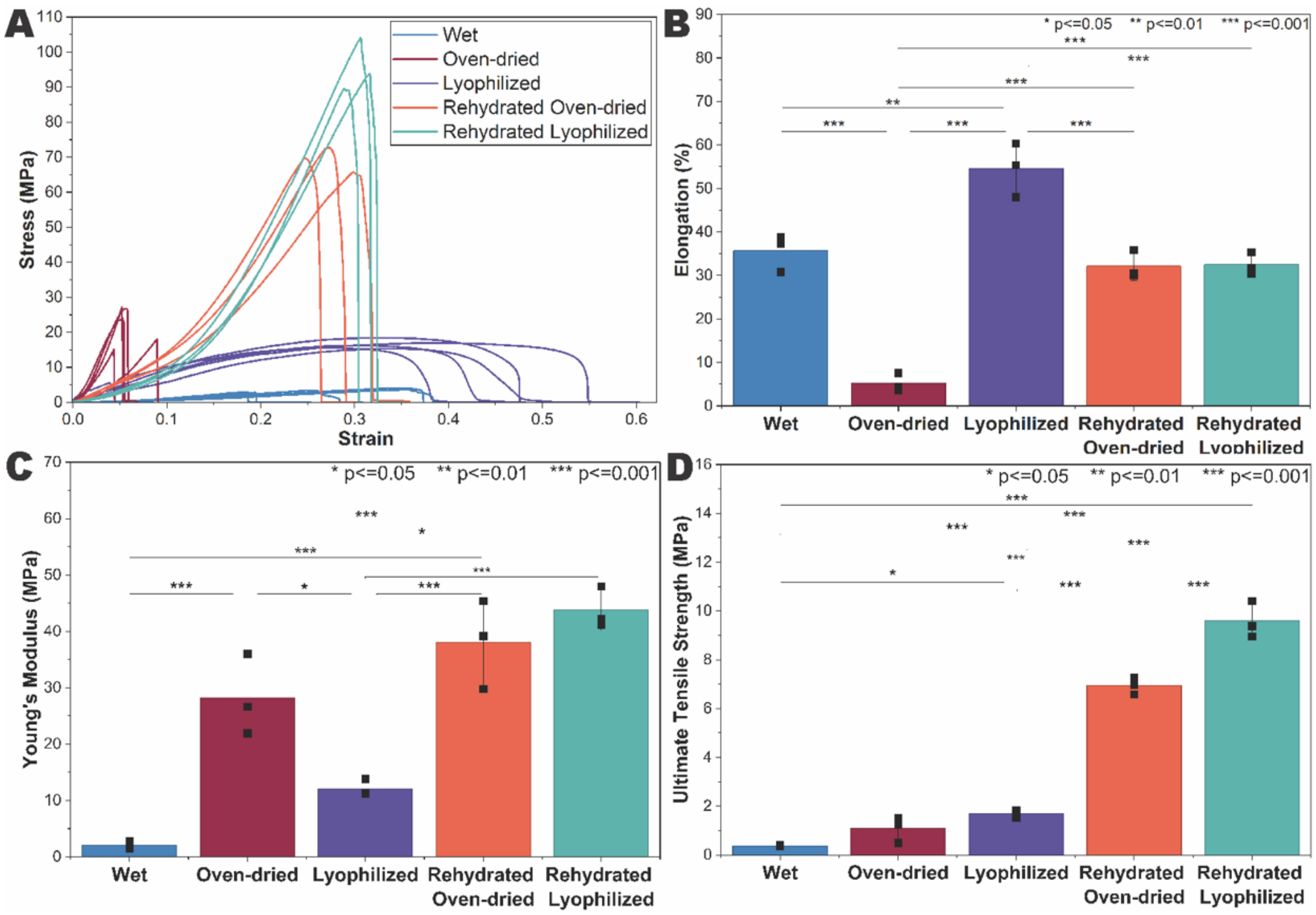

Figure S1. **(A)** Stress-strain curves, **(B)** elongation, **(C)** Young's modulus, and **(D)** ultimate tensile strength of various kombucha-derived non-wovens. Error bars denote one standard deviation (n = 3).

Table S3. Viscoelastic Response of Kombucha-Derived Non-Wovens.

**Sample Name**	**G′ at 1 rad s^{-1}**	**LVR Limit in Strain Sweep (%)**
Wet	1.5×10^5	0.05
Oven-dried	4.6×10^4	0.2
Lyophilized	3.4×10^5	0.4

Table S4. Benchmarking Mechanical Properties of Cellulose Non-Wovens.

Sample Details		Tensile Strength (MPa)	Young's Modulus (MPa)	Ref
**Lyophilized**	**Kombucha-derived non-wovens**	**14.36 ±0.9**	**40.84 ±6.7**	**This work**
	Bacterial cellulose membrane (*Komagataeibacter sp.*)	9.2 ±3.5	68.6 ±5.2	[1]
**Oven-dried**	**Kombucha-derived non-wovens**	**2.54 ±0.3**	**31.99 ±5.5**	**This work**
	Unmodified cellulose non-woven	4.3 ±0.5	9.0 ±1.6	[2]
	Wet unmodified cellulose non-woven	2.2 ±0.1	8.2 ±0.4	[2]
	Bacterial cellulose biofilm	2.78	27.91	[3]
	Bacterial cellulose membrane	8.126	62.776	[4]
	Crude dry bacterial cellulose	11.6 ±0.8	180.3 ±10.6	[5]
**Wet**	**Kombucha-derived non-wovens**	**1.66 ±0.3**	**10.27 ±8.4**	**This work**
	Bacterial cellulose hydrogel (*G. hansenii* CGMCC 3917)	0.26	0.005	[6]
	Bacterial cellulose membrane (*Komagataeibacter sp.* ATCC 700178)	0.15 ±0.08	1.10 ±0.4	[7]
	Bacterial cellulose membrane (*Komagataeibacter sp.* ATCC 10245)	0.36 ±0.08	2.87 ±1.3	[7]
	Bacterial cellulose membrane (*Komagataeibacter sp.* ATCC 23769)	0.12 ±0.04	1.26 ±0.7	[7]
	Bacterial cellulose membrane (*Komagataeibacter sp.* NBRC 13693)	0.62 ±0.2	3.08 ±0.7	[7]
	Bacterial cellulose membrane (*Komagataeibacter sp.* ATCC 53524)	0.68 ±0.1	5.56 ±2.3	[7]
	Bacterial cellulose membrane (*Komagataeibacter sp.* KTH 5655)	0.62 ±0.2	3.83 ±1.1	[7]
	Bacterial cellulose membrane	4.770	13.305	[4]
	Bacterial cellulose membrane (pH 5.0 Glycerol culture)	1.70	3.70	[7]
	Bacterial cellulose membrane (Glucose culture)	0.12	1.26	[6]
	Bacterial cellulose hydrogel	0.12-0.68	1.10-5.56	[8]
	Bi-networked cellulose nanohydrogel (clicked chemical cross-linking)	0.43	10	[9]
	Bacterial cellulose membrane	1 ±0.0	10 ±0.0	[8]
	Kombucha SCOBY	0.27 ±0.08	1.09 ±0.4	[11]

	Crude fresh bacterial cellulose	1.2 ±0.2	3.1 ±0.5	[5]
	non-dried bacterial cellulose	1.18 ± 1.01		
**Rehydrated Oven-dried**	**Kombucha-derived non-wovens**	**6.95 ±0.3**	**38.09 ±7.9**	**This work**
	Crude bacterial cellulose	127.2 ±13.9	25.1 ±1.8	[5]
	25 °C convection-dried, then soaked	17.40 ± 3.68		[12]
	40 °C convection-dried, then soaked	16.98 ± 4.34		[12]
	80 °C convection-dried, then soaked	8.49 ± 5.47		[12]
	105 °C convection-dried, then soaked	5.23 ± 1.59		[12]
**Rehydrated Lyophilized**	**Kombucha-derived non-wovens**	**9.58 ±0.7**	**43.83 ±3.7**	**This work**
	Freeze-dried, then soaked	10.04 ± 2.32		[12]

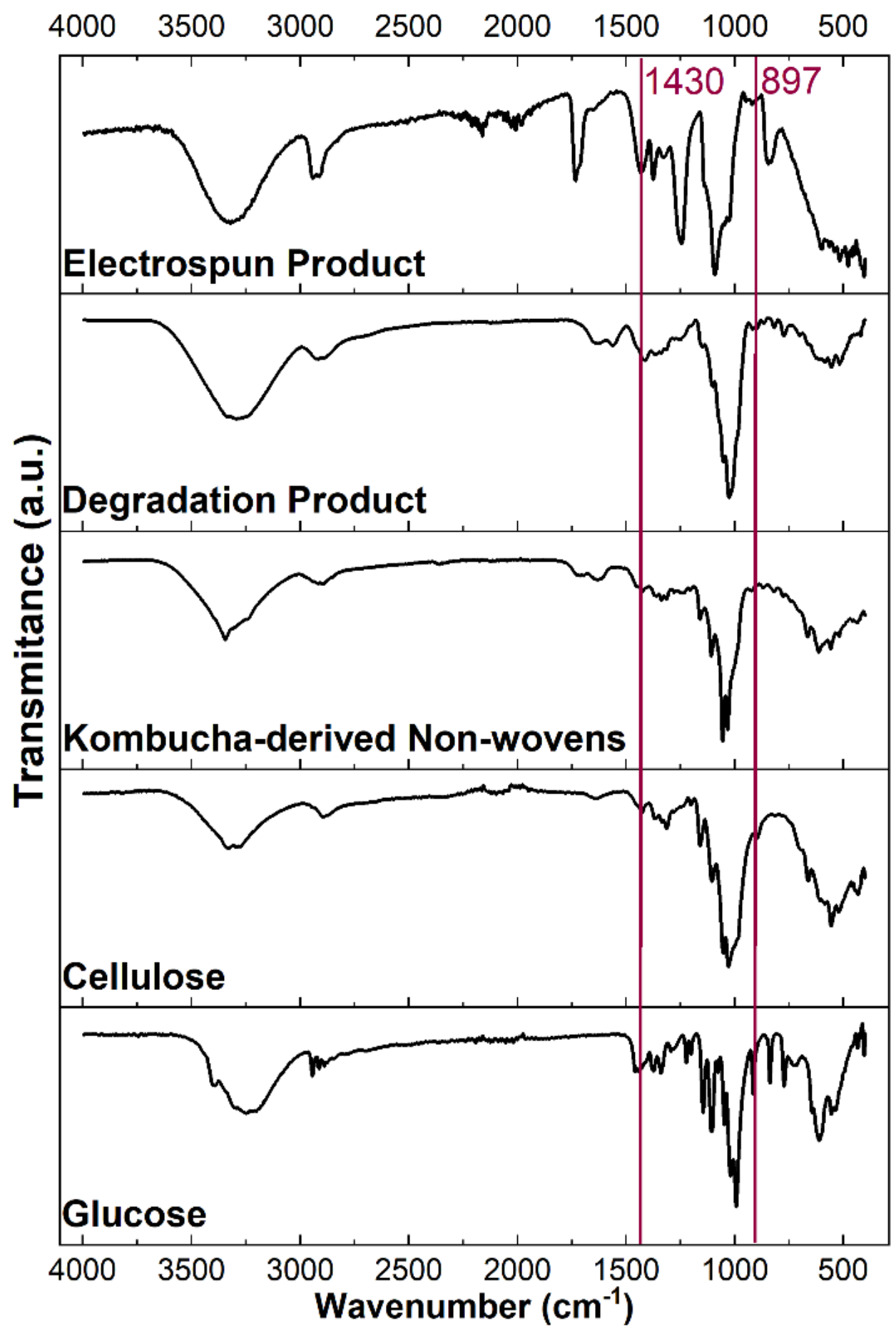

Figure S2. FTIR spectra of glucose, cellulose, kombucha-derived cellulose non-wovens, enzymatic degradation product, and electrospun fibers. The peaks at ~1430 cm^{-1} and ~897 cm^{-1} indicate cellulose-associated bands assigned to CH_2 bending and the β-(1→4)-glycosidic linkage, respectively.

Table S5. FTIR Peak Assignments for Cellulose, Glucose, and Kombucha-Derived Samples.

**Wavenumber** (cm^{-1})	**Assignment**	**Glucose**	**Cellulose**	**Kombucha Non-wovens**	**Degradation Product**	**Electrospun Product**
~3330	O-H stretching[13]	Yes	Yes	Yes	Yes	Yes
~2900	C-H stretching[14]	Yes	Yes	Yes	Yes	Yes
~1430	CH_2 bending[14]	No	Yes	Yes	Yes	Yes
~897	β-(1→4) glycosidic vibration[14]	No	Yes	Yes	Yes	Yes
1655 / 1540	Amide I / II[15]	No	No	Yes	No	No
1150-1000	C-O / C-C stretching[14]	Yes	Yes	Yes	Yes	Yes

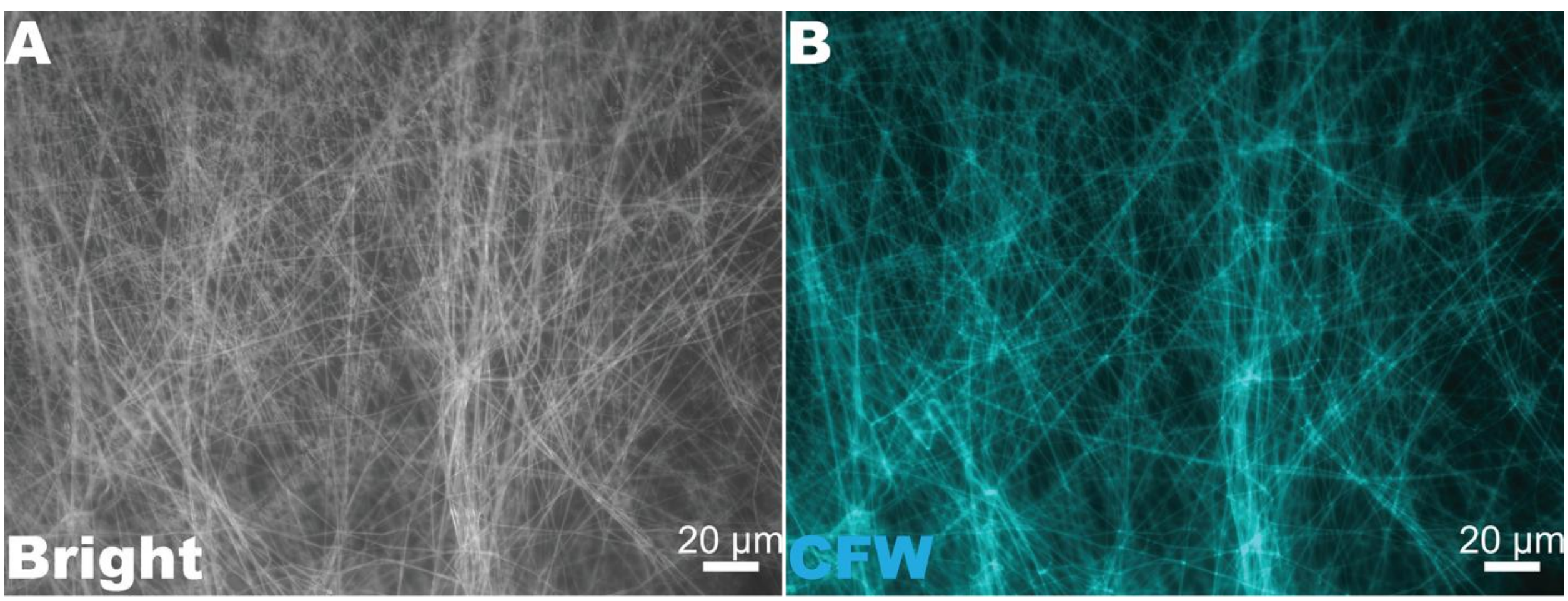

Figure S3. **(A)** Brightfield and **(B)** fluorescence images of electrospun fibers prepared from kombucha-derived hydrolysis products. Scale bars: 20 µm.

REFERENCES

(1) Fatima, A.; Ortiz-Albo, P.; Neves, L. A.; Nascimento, F. X.; Crespo, J. G. Biosynthesis and Characterization of Bacterial Cellulose Membranes Presenting Relevant Characteristics for Air/Gas Filtration. *J. Membr. Sci.* **2023**, *674*, 121509. https://doi.org/10.1016/j.memsci.2023.121509.

(2) Jalvo, B.; Aguilar-Sanchez, A.; Ruiz-Caldas, M.-X.; Mathew, A. P. Water Filtration Membranes Based on Non-Woven Cellulose Fabrics: Effect of Nanopolysaccharide Coatings on Selective Particle Rejection, Antifouling, and Antibacterial Properties. *Nanomaterials* **2021**, *11* (7), 1752. https://doi.org/10.3390/nano11071752.

(3) Sederavičiūtė, F.; Domskienė, J.; Baltina, I. Influence of Drying Temperature on Tensile and Bursting Strength of Bacterial Cellulose Biofilm /. *Mater. Sci.* **2019**, *25* (3), 316–321. https://doi.org/10.5755/j01.ms.25.3.20764.

(4) Bodea, I. M.; Beteg, F. I.; Pop, C. R.; David, A. P.; Dudescu, M. C.; Vilău, C.; Stănilă, A.; Rotar, A. M.; Cătunescu, G. M. Optimization of Moist and Oven-Dried Bacterial Cellulose Production for Functional Properties. *Polymers* **2021**, *13* (13), 2088. https://doi.org/10.3390/polym13132088.
(5) Pogorelova, N.; Rogachev, E.; Digel, I.; Chernigova, S.; Nardin, D. Bacterial Cellulose Nanocomposites: Morphology and Mechanical Properties. *Materials* **2020**, *13* (12). https://doi.org/10.3390/ma13122849.
(6) Chen, S.-Q.; Lopez-Sanchez, P.; Wang, D.; Mikkelsen, D.; Gidley, M. J. Mechanical Properties of Bacterial Cellulose Synthesised by Diverse Strains of the Genus *Komagataeibacter*. *Food Hydrocoll.* **2018**, *81*, 87–95. https://doi.org/10.1016/j.foodhyd.2018.02.031.
(7) Chen, S.-Q.; Lopez-Sanchez, P.; Wang, D.; Mikkelsen, D.; Gidley, M. J. Mechanical Properties of Bacterial Cellulose Synthesised by Diverse Strains of the Genus *Komagataeibacter*. *Food Hydrocoll.* **2018**, *81*, 87–95. https://doi.org/10.1016/j.foodhyd.2018.02.031.
(8) Yuan, B.; Ritzoulis, C.; Chen, J. Extensional and Shear Rheology of a Food Hydrocolloid. *Food Hydrocoll.* **2018**, *74*, 296–306. https://doi.org/10.1016/j.foodhyd.2017.08.019.
(9) Rothe, H.; Rost, J.; Kramer, F.; Alkhatib, Y.; Petzold-Welcke, K.; Klemm, D.; Fischer, D.; Liefeith, K. Bacterial Nanocellulose: Reinforcement of Compressive Strength Using an Adapted Mobile Matrix Reservoir Technology and Suitable Post-Modification Strategies. *J. Mech. Behav. Biomed. Mater.* **2022**, *125*, 104978. https://doi.org/10.1016/j.jmbbm.2021.104978.
(10) Bodea, I. M.; Beteg, F. I.; Pop, C. R.; David, A. P.; Dudescu, M. C.; Vilău, C.; Stănilă, A.; Rotar, A. M.; Cătunescu, G. M. Optimization of Moist and Oven-Dried Bacterial Cellulose Production for Functional Properties. *Polymers* **2021**, *13* (13), 2088. https://doi.org/10.3390/polym13132088.
(11) Chaussé, J.; Girard, V.-D.; Perron, T.; Challut, T.; Vermette, P. Characterization of Bacterial Cellulose Produced by the KomEt Strain Isolated from a Kombucha SCOBY. *Biocatal. Agric. Biotechnol.* **2024**, *58*, 103172. https://doi.org/10.1016/j.bcab.2024.103172.
(12) Stanisławska, A.; Staroszczyk, H.; Szkodo, M. The Effect of Dehydration/Rehydration of Bacterial Nanocellulose on Its Tensile Strength and Physicochemical Properties. *Carbohydr. Polym.* **2020**, *236*, 116023. https://doi.org/10.1016/j.carbpol.2020.116023.
(13) Cichosz, S.; Masek, A. IR Study on Cellulose with the Varied Moisture Contents: Insight into the Supramolecular Structure. *Materials* **2020**, *13* (20), 4573. https://doi.org/10.3390/ma13204573.
(14) Wang, S.-S.; Han, Y.-H.; Ye, Y.-X.; Shi, X.-X.; Xiang, P.; Chen, D.-L.; Li, M. Physicochemical Characterization of High-Quality Bacterial Cellulose Produced by Komagataeibacter Sp. Strain W1 and Identification of the Associated Genes in Bacterial Cellulose Production. *RSC Adv.* **2017**, *7* (71), 45145–45155. https://doi.org/10.1039/C7RA08391B.
(15) Fuller, M. E.; Andaya, C.; McClay, K. Evaluation of ATR-FTIR for Analysis of Bacterial Cellulose Impurities. *J. Microbiol. Methods* **2018**, *144*, 145–151. https://doi.org/10.1016/j.mimet.2017.10.017.
